# Updating “processing and consolidation of open data on public procurement in France (2015–2023)” with daily refresh

**DOI:** 10.1016/j.dib.2025.112362

**Published:** 2025-12-08

**Authors:** Adrien Deschamps

**Affiliations:** Avignon Université, Laboratoire LBNC, 74 Rue Louis Pasteur, 84029 Avignon, France

**Keywords:** Open data, E-procurement, Corruption, Green public procurement

## Abstract

Public procurement transparency is a major policy concern worldwide. It is expected to improve the evaluation of procurement regulations, prevent corruption, and foster competition, given the significant weight of public contracts in government spending. For instance, in 2023 the European Union implemented the *eForms* standard to harmonize procurement notices across its member states. This article presents an updated version of a previous dataset that collected and enriched open data on French public procurement, which could no longer be maintained after the adoption of *eForms*. The new version not only updates the data from 2024 onward but also introduces two major improvements. First, it is automatically refreshed on a daily basis. Second, it relies on a faster and more reliable method for identifying the public and private actors involved in procurement. This article therefore provides a methodology for a continuous and accurate centralization and enhancement of procurement notices. The resulting dataset is intended to benefit researchers, policymakers, businesses, and public buyers alike.

Specifications Table

**Table:** 

Subject	Microeconomics
Specific subject area	The dataset results from the daily processing and consolidation of French public contract notices augmented with individual information on economic agents.
Data format	.parquet and .csv
Type of data	Table
Data collection	Calls for tenders and award notices are downloaded from the website of the official journal for French public procurement. The content of the notices is then filtered and processed. After that, missing identifiers of firms and contracting authorities are estimated, using an online request API. Finally, the dataset is consolidated by importing individual characteristics of firms and authorities, including their geolocation.
Data source location	The official website for public procurement notices (BOAMP): https://www.boamp.fr/pages/donnees-ouvertes-et-api/ Data on organizations and their location: https://www.data.gouv.fr/fr/datasets/base-sirene-des-entreprises-et-de-leurs-etablissements-siren-siret/ https://www.data.gouv.fr/fr/datasets/geolocalisation-des-etablissements-du-repertoire-sirene-pour-les-etudes-statistiques/ Data on municipal federations: https://www.insee.fr/fr/information/2510634
Data accessibility	Repository name: BeauAMP-Daily: continuous processing and consolidation of French public procurement open data Data identification number: 10.5281/zenodo.17187786 Direct URL to data: https://zenodo.org/records/17187786
Related data article	A. Deschamps, L. Potin. Processing and consolidation of open data on public procurement in France (2015–2023). *Data in Brief* (2025), 10.1016/j.dib.2025.111277
Related research article	None

## 1 Value of the Data

• The dataset enables the aggregation and analysis of information that was previously scattered across disparate documents. It also enhances the content of the original notices by addressing the inconsistencies in their content, for example by categorizing the content and standardizing the weights of the award criteria (i.e., the different aspects of an offer evaluated by the contracting authority). The dataset also augments the original data by adding detailed descriptions of awarded firms and contracting authorities (e.g., staff size, legal status, creation date, main activity, geolocation) after estimating their official identifiers.
• This updated version significantly improves the initial version of the dataset [1]. While the original version (covering notices published between 2015 and 2023) had to be manually updated, this updated version is refreshed and automatically uploaded every day [2]. Additionally, as the format of procurement notices changed in January 2024, the scripts that generate the updated dataset are adapted to the new format. The identification of awarded firms and contracting authorities in the updated dataset relies on a new algorithm based on online requests, which reduces computational time and provides more reliable information on the individual characteristics of economic agents compared to the one of Potin et al. [3].

## 2 Data Description

Daily data can be found on the following repository (in French): https://www.data.gouv.fr/datasets/base-etendue-amelioree-et-unifiee-des-annonces-des-marches-publics/. For academic research purpose, annual data is published on Zenodo in English, both in CSV and Parquet format.

**Fig. 1:**
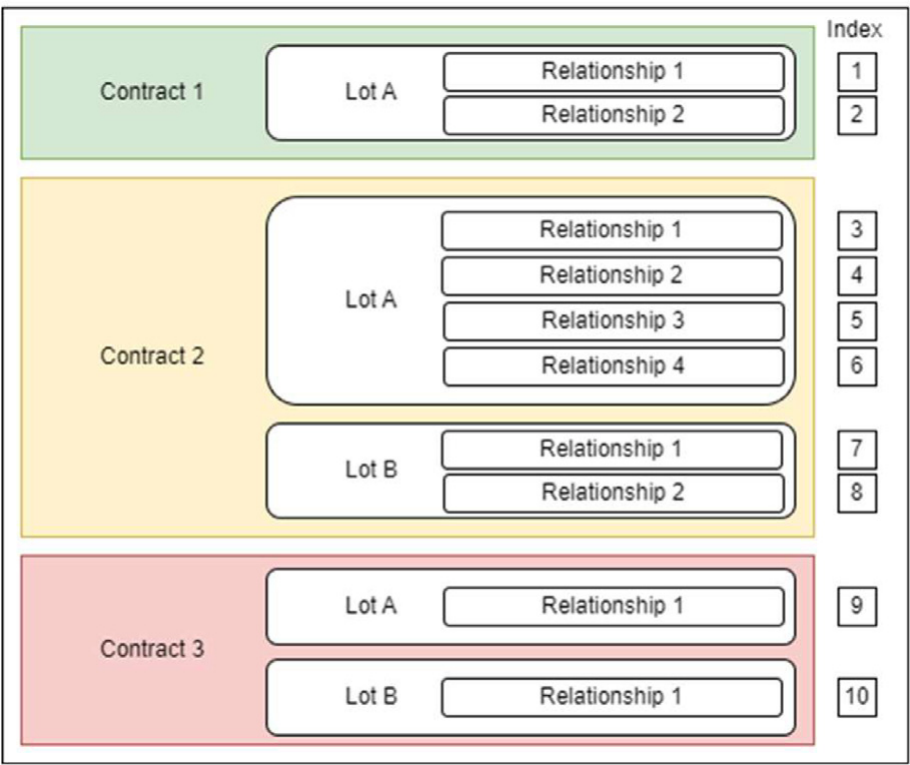
Diagram of the dataset structure.

The dataset comprises 72,040 rows and 88 columns that account for 88 variables describing contracts, the outcome of award procedures, contracting authorities, and awarded firms. The dataset encapsulates 24,293 contracts awarded in 2024 which value exceeds European advertising thresholds. As highlighted by Fig. 1, each row corresponds to a contractual relationship between a purchasing authority and an awarded firm. Note that a given contract (identified by the variable “ID_BOAMP_AWARD”) can be split into different lots (identified by the variable “LOT_ID”).

As is in the original data paper [4], there are too many variables to provide an exhaustive list in this paper. For the sake of clarity, an Excel file with the name, meaning, and availability of each variable is attached to this article. Additionally, Table 1 lists the variables that appeared in the updated version compared to the initial one, and conversely the variables that are no longer available following the *eForms* implementation. In addition, note that all the variables referring to specific agencies of contracting authorities and awarded firms in the original dataset (variable names that include “_AGENCY”) have been removed, as the updated dataset no longer considers specific agencies (the new data focuses on SIREN identifiers and overlooks SIRETs for the sake of simplicity).

**Table 1:** Variables that have been removed or added after the dataset update.

New variables		Removed variables	
Name	Definition	Name	Definition
CONTRACT_START	The day the contract performance begins.	SUBCONTRACTING	Indicates whether the awarded firm resorts to subcontracting.
EU_FUNDED	Indicates whether the European Union has supported the purchase.	NUMBER_NON_EU_OFFERS	Indicates the number of firms located outside the EU that participated in the award procedure.
FUNDS_NAME	The name of the European funds.	NUMBER_EU_OFFERS	Indicates the number of firms located in the EU that participated in the award procedure.
MAX_TOTAL_VALUE_ FRAMEWORK_AGGREMENT	The maximum value of the framework agreement.	MIN_OFFER	Indicates the lowest bid the contracting authority received.
SME_FRIENDLY	Indicates whether the public authority has made a specific effort to support SME participation.	MAX_OFFER	Indicates the highest bid the contracting authority received.
STRATEGIC_ENVIRONMENTAL	Indicates whether the contract is part of a broader green strategy.	ON_BEHALF	Indicates whether the contract was made by an authority for another public institution.
STRATEGIC_SOCIAL	Indicates whether the contract is part of a broader social strategy.		

## 3 Experimental Design, Materials and Methods

As for the original version of the dataset, remind that French public contracts which value exceed some legal thresholds must be published on the BOAMP official bulletin. First, a contract notice is issued, acting as a call for tenders that describes the contract and the rules of the award procedure. After a certain advertising period, the contracting authority selects one or several firms. Its decision is communicated in an award notice.

The following GitHub repository contains nine Python scripts used to download, process, and consolidate the information contained in contract and award notices published on the BOAMP website since January 2024 (when the contract value exceeds European thresholds, i.e. when the contract must also be published on the official journal of the European Union and follow the *eForms* format): https://github.com/AdrienDeschampsAU/BeauAMP-Daily.

### 3.1 Data download and processing

**Fig. 2:**
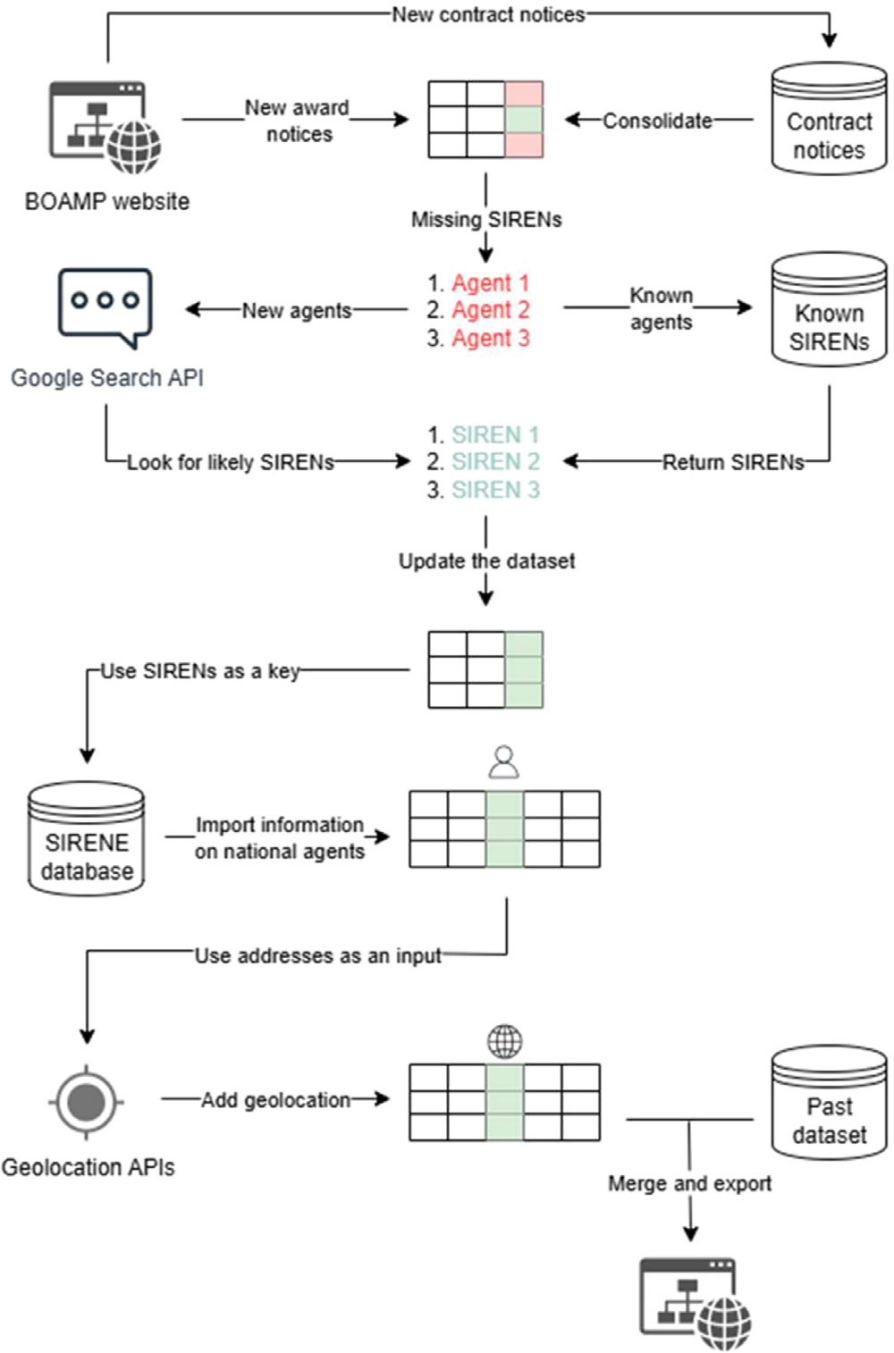
Summary of the pipeline that generates the dataset.

As encapsulated by Fig. 2, contract and award notices that were published on the BOAMP website the day before are downloaded with a dedicated API (see the file “download.py”). Award notices are immediately processed into a tabular format (see “processing.py”), while contract notices are saved to be processed when the corresponding award notice is published.

### 3.2 Agent identification

SIREN is a 9-digit code assigned to every legal entity in France. The SIREN of contracting authorities and awarded firms is supposed to be mentioned in the notices, but it is available in only 60 % of the notices for contracting authorities and 30 % for awarded firms. After data processing, missing SIRENs are exported to a separate file (see “missing_sirens.py”). There are two different scenarios for estimating these identifiers (see “sirenisation.py”). If the stated name and address of the agent (either a contracting authority or an awarded firm) have previously been available or estimated, the corresponding SIREN is assigned to the agent. If the name and address do not match any known agent, Google Custom Search JSON API is used with requests such as “{stated name} + {city in the stated address}” on a public directory on French organizations (https://annuaire-entreprises.data.gouv.fr) to estimate SIRENs. Note that compared to the original machine learning algorithm [5], this new strategy requires less computational time, since there is no need to look for matches in a large database, but it is more costly, as API requests are charged. It appears to be slightly more accurate than the initial machine learning approach. While the methodology developed by Potin et al. [5] could find the correct SIREN of contracting authorities and awarded firms with an approximate success rate of 80 % and 70 % respectively, API requests return the correct SIREN in 95 % of the cases for contracting authorities and 80 % for firms (based on two random sets of 200 unique stated names and addresses with no associated SIREN, after checking manually the actual SIRENs).

### 3.3 Consolidation

After adding the estimated identifiers to the dataset (see “siren_import.py”), SIRENs are used to import individual characteristics from the SIRENE official database (see “consolidation.py”). Firms and contracting authorities are geolocated based on the address mentioned in award notices. When the stated address is in France, geolocation resorts to the BAN (*Base Adresse Nationale*) public API (see “national_geolocation.py”), while addresses located in the rest of the world are geolocated with the Nominatim API (see “world_geolocation.py”). Finally, daily data is merged with the past dataset (see “final_merger.py”).

## Limitations

The availability of variables – including meaningful ones such as the contract estimated value – can be limited, as civil servants are not required to mention every aspect of the contract in the notices they fill. Nothing can be done to recover this information. Although the accuracy in the estimation of agent identifiers has slightly increased compared to the previous version, there is still a risk that the returned SIREN may not correspond to the right contracting authority or awarded firm. Hence, the variables that describe the contracting parties may sometimes not reflect their real features. Finally, the updated dataset focuses on contracts which value exceed European advertising thresholds as they are the only ones that follow the *eForms* format, while the original data also included contracts with lower values (although the available information in these contracts was quite poor as there was no standardized format for them).

## Ethics Statement

The author has read and follows the ethical requirements for publication in Data in Brief and confirms that the current work does not involve human subjects, animal experiments, or any data collected from social media platforms.

## CRediT Author Statement

The sole author of this work is Adrien Deschamps.

## Data Availability

ZenodoBeauAMP-Daily: continuous processing and consolidation of French public procurement open data (Original data).

## Appendix Supplementary materials

Image, application 1
